# An Augmented Reality-Based Interaction Scheme for Robotic Pedicle Screw Placement

**DOI:** 10.3390/jimaging8100273

**Published:** 2022-10-06

**Authors:** Viktor Vörös, Ruixuan Li, Ayoob Davoodi, Gauthier Wybaillie, Emmanuel Vander Poorten, Kenan Niu

**Affiliations:** 1 Robot-Assisted Surgery Group, Department of Mechanical Engineering, KU Leuven, Celestijnenlaan 300, 3000 Leuven, Belgium; 2 Healthcare Division, Barco NV, Beneluxpark 21, 8500 Kortrijk, Belgium

**Keywords:** augmented reality, robotic control, pedicle screw placement, AR interaction

## Abstract

Robot-assisted surgery is becoming popular in the operation room (OR) for, e.g., orthopedic surgery (among other surgeries). However, robotic executions related to surgical steps cannot simply rely on preoperative plans. Using pedicle screw placement as an example, extra adjustments are needed to adapt to the intraoperative changes when the preoperative planning is outdated. During surgery, adjusting a surgical plan is non-trivial and typically rather complex since the available interfaces used in current robotic systems are not always intuitive to use. Recently, thanks to technical advancements in head-mounted displays (HMD), augmented reality (AR)-based medical applications are emerging in the OR. The rendered virtual objects can be overlapped with real-world physical objects to offer intuitive displays of the surgical sites and anatomy. Moreover, the potential of combining AR with robotics is even more promising; however, it has not been fully exploited. In this paper, an innovative AR-based robotic approach is proposed and its technical feasibility in simulated pedicle screw placement is demonstrated. An approach for spatial calibration between the robot and HoloLens 2 without using an external 3D tracking system is proposed. The developed system offers an intuitive AR–robot interaction approach between the surgeon and the surgical robot by projecting the current surgical plan to the surgeon for fine-tuning and transferring the updated surgical plan immediately back to the robot side for execution. A series of bench-top experiments were conducted to evaluate system accuracy and human-related errors. A mean calibration error of 3.61 mm was found. The overall target pose error was 3.05 mm in translation and $1.12^{\circ }$ in orientation. The average execution time for defining a target entry point intraoperatively was 26.56 s. This work offers an intuitive AR-based robotic approach, which could facilitate robotic technology in the OR and boost synergy between AR and robots for other medical applications.

## 1. Introduction

Annually, 266 million individuals suffer from spinal diseases worldwide, with an incident rate of 5.7% in Europe [1]. Spinal diseases severely reduce the quality of the patient’s life and put a huge burden on the current healthcare system. Pedicle screw placement (PSP) is one of the most critical surgical steps in spine fusion surgery as a pedicle screw needs to be precisely inserted into a narrow canal inside the vertebra. Screws are placed in close proximity to the spinal canal. Therefore, a small mispositioning of the screw may cause severe complications, e.g., breaching and damage to the spinal cord. Misplacement may lead to a dural lesion, nerve root, or spinal cord injury [2]. Conventional free-hand PSP surgery only has a success rate of approximately 90%. In complex cases, the failure rate may reach up to 14% [3]. In this context, the ability to adjust intraoperative planning on-site is very important. The surgeon can monitor the entire surgical procedure and lower the amount and chance of misalignment.

In conventional PSP, screw positioning is done by free-hand operation. In open surgery, the surgeon inserts screws based on bone landmarks. Traditionally, this happens without the assistance of image guidance. Experts rely on rich experiences to map the bony landmarks to the pre-operatively planned trajectory. Recently, computer-assisted navigation with medical imaging became popular in the operating room (OR) as it offers additional guidance for the surgeon. C-arm fluoroscopy is often used in PSP as it provides a live, continuous X-ray image that is visualized on a display. This intraoperative imaging provides information on how the drill is positioned with respect to the vertebra. Based on this image, the surgeon can update the instrument’s position. In-vivo studies showed that fluoroscopy-based guidance improves the pedicle screw insertion accuracy to 93.9% among 2003 cases [4]. Though achieving better results, existing intraoperative navigation systems for PSP still face several challenges. Hand–eye coordination remains difficult as the surgeon needs to look at a monitor. The shift of focus away from the operating site and the mapping of the guidance from a 2D image to a 3D anatomy puts a large mental load on the surgeon. Additionally, the related surgical planning is normally devised before surgery and, thus, cannot be easily adapted to unexpected misalignment that happens during the surgery.

Recently, some navigation systems based on augmented reality (AR) or mixed reality (MR) were proposed for spine surgery [5,6,7,8]. Muller et al. [9] evaluated the surgical accuracy of pedicle screw navigation using an HMD via 3D intraoperative fluoroscopy. The proposed system showed an accuracy of 3.4±1.6 mm transitional error and $4.3\pm 2.3^{\circ }$ rotational error. However, radiative intraoperative navigation (i.e., fluoroscopy) was still involved to assist the drilling procedure. Gibby et al. [6] utilized an AR-based head-mounted device for free-hand percutaneous PSP and achieved a 4.69 mm mean distance on an opaque lumbar model. Although omitting radiation, the system performance was still influenced by manual operation, which led to higher errors. Liu et al. [7] also proposed an intraoperative CT-based AR-guided drilling system to assess the feasibility and accuracy of percutaneous pedicle screw placements compared to a radiography-guided method. The accuracy of the AR-guided system was 94%, which is more acceptable than the radiography-guided approach. However, this work leveraged registration by using AR and pre-operative CT scans. The interaction and the ability to adjust pre-operative planning have not been investigated. Overall, AR-based guidance could improve PSP precision in a laboratory setting [8]. However, as far as the authors are aware, AR navigation was only implemented and conducted with free-hand pedicle screw placement. The success rate was also influenced by free-hand manipulation and drilling. To improve surgical accuracy, a stable and reliable approach is crucial to assist the surgeon to perform the drilling procedure.

Robot-assisted approaches have been widely investigated to improve the feasibility and repeatability of screw placement [10,11,12]. Such robotic approaches could achieve superior levels of precision and avoid surgeon fatigue and tremors. The use of the robotic approach was shown to improve the performance and to increase the accuracy of PSP by 58% [10,13]. Currently, existing AR-based navigation systems have not been fully integrated with robotic-assisted systems. Furthermore, there are only a few studies that have integrated robot assistance systems with AR-based guidance for surgical applications. Ferraguti et al. [14] proposed AR-based robotic percutaneous nephrolithotomy to improve the intervention performance and reduce the learning curve. As a result, the average translation error of intervention was 15.18±12.75 mm while the execution time was reduced from 25.89 to 19.56 s among 11 users. For PSP, Tu et al. [15] proposed a robotic-assisted ultrasound (US) system with mixed reality to construct a virtual physical fusion surgical scene. The system yields an average deviation of 1.04±0.27 mm for cervical pedicle screw placement. Despite the promising results, the HoloLens was only employed to visualize the preoperative planning and guarantee the correctness, rather than to directly guide the intraoperative screw placement. Existing AR-based robotic drilling systems need to improve their accuracy and robustness, and their performances need to be quantitatively assessed.

The paper introduces an intuitive interactive control scheme for human–robot interaction (HRI) exploiting AR to guide a surgical robotic arm for pedicle screw placement. The system allows the user to visualize and adjust the surgical plan intraoperatively via the AR scene. The immersive user interface allows the user to monitor the robot’s execution and intervene. Hereto, the operator can co-manipulate the robot to perform surgical actions, such as positioning the robot, drilling, and retraction.

## 2. Materials and Methods

**Figure 1:**
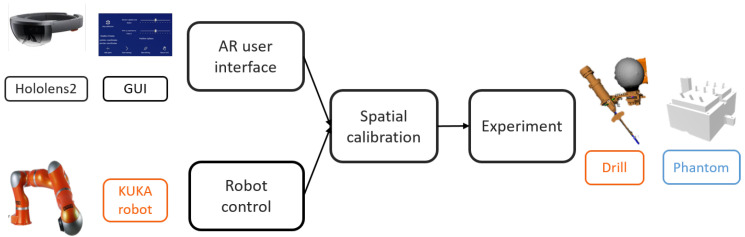
The framework for the proposed system, including the main steps: AR user interface, robot control, spatial calibration, and experiments on the phantom.

Figure 1 illustrates the proposed system, consisting of (1) a head-mounted display (HMD) (Microsoft HoloLens2) in which an AR application was developed to offer AR visualization and interaction; (2) a lightweight robotic arm (KUKA Robot LWR) with a custom-designed drilling platform mounted on the robot’s end-effector, and (3) a PC work station (Intel i7, CPU @2.6 GHz, 64G RAM) with a WiFi router (TP-link Archer C80). Since no external tracking device was used, it suffices to define only the HoloLens {H}, the robot {R}, the drill {D}, and the phantom {Ph} coordinate frames.

**Figure 2:**
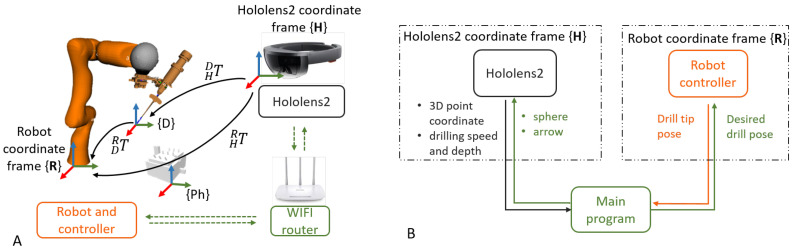
(**A**) System overview of the involved transformations and coordinate frames. The notation ${}_{A}^{B}T$ denotes the transformation from coordinate frame A to B. (**B**) A schematic overview of the functional modules of the proposed system.

The communication between the robot controller and the robotic arm was achieved using the robot operating system (ROS). To ensure real-time control, OROCOS (Open Robot Control Middleware) and eTaSL (expression graph-based Task Specification Language) were utilized [16]. A User Datagram Protocol (UDP) connection was established between the HoloLens and the PC, allowing bidirectional data transmission. The transmitted data included 3D poses and control parameters, such as drilling speed and depth. Thanks to wireless communication, the user was not restricted in his/her motion. Figure 2 shows the overview of the system.

### 2.1. Robot Control

In this paper, admittance and position control were used in two different situations. The admittance controller was used to interact with the user’s motion during the calibration and registration. In this mode, the user could directly grab the drilling platform attached to the robot and co-manipulate it to the desired pose. This concept was based on mechanical admittance as presented in [17] where the target robot end effector velocity was computed from the force exerted at the end effector.

The position control was implemented to perform the automatic positioning and drilling after the calibration. The eTaSL optimization core sent the desired joint angle $q_{d}$ as input to the KUKA controller and the robot executed the corresponding motion. The motion speed was set as 2 mm/s. Meanwhile, the desired drilling depth was set as 50 mm with a 1000 rpm drilling speed.

### 2.2. Augmented Reality User Interface

**Figure 3:**
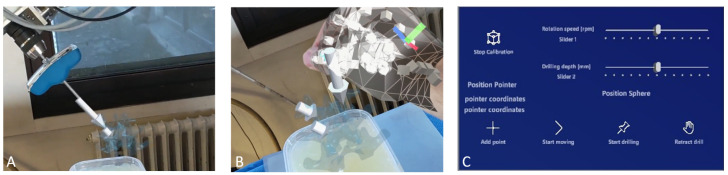
(**A**) The robot aligns the drill with the target arrow in AR; (**B**) Online adjustment of the surgical plan: a user can move and define the desired drilling position and orientation in the AR scene. (**C**) The GUI running in the HoloLens. By using the GUI panel in AR, the control command can also be sent to the robot controller for execution. The user could press the buttons or grab and move the sliders using hand gestures.

A dedicated AR application was developed in Unity to establish the interface for the human–robot interaction, using a HoloLens2 HMD. This HRI was achieved by visualizing the interactive 3D AR cues, such as spheres and arrows, and a dedicated graphical user interface (GUI). The interaction was based on the built-in hand gesture and voice commands of the HoloLens, as the user could grab and manipulate the AR cues, or interact with the buttons and sliders on the AR GUI using his/her fingers. Such interaction omits the need for a physical controller, e.g., mouse, keyboard, or control panel. With the GUI, the user was able to (1) add a new entry point, (2) start/stop motion, (3) control the drill and (4) set drilling parameters, such as drilling depth and rotation speed, as shown in Figure 3C.

The desired pose of the drill tip $p_{H}$ could be defined in {H} by placing a virtual arrow overlaying with real-world anatomy. This information was converted to the robot frame using ${}_{H}^{R}T$, the transform from {H} to {R}, as:

(1) $$p_{R}{=}_{H}^{R}T\cdot p_{H}$$

where $p_{H}$ and $p_{R}$ are the desired poses in {H} and {R}, respectively. Then, the robot was controlled to reach the desired pose using position control. This touch-free control method is appealing for surgical use since there is no need to sterilize a physical controller and the surgeon does not need to shift his/her focus between the surgical site and a display. Figure 3 shows an example of placing the virtual arrow and adding new drilling poses via the GUI.

### 2.3. Spatial Calibration

To be able to convert the pose of the virtual arrow to the robot frame, the transformation matrix between the HoloLens coordinate frame {H} and the robot coordinate frame {R} was determined by spatial calibration. The system required no external tracking device during the calibration since a method relying on the HoloLens functionality is proposed. In general, when the HoloLens launches an application, it determines a fixed coordinate frame by sensing the surrounding environment. As the user moves around, the established reference coordinate system remains stationary in its initial position. Thus this frame {H} did not move anymore, even when the user was moving. After each reboot of the HoloLens, this frame is renewed so an efficient way to register this frame with respect to the robot frame is needed.

**Figure 4:**
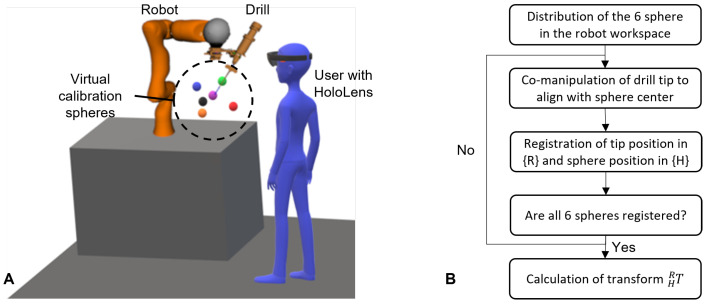
(**A**) The layout of the calibration procedure. The colored spheres are the AR calibration points seen by the user through the HoloLens. (**B**) The flowchart of the calibration procedure.

For this, the spatial calibration in this work proposes the use of six virtual spheres that were rendered in the HoloLens scene {H}. During the calibration procedure, the user first grabbed each sphere using hand gestures and moved them to the robot workspace, such that they were in a uniform spatial distribution, as shown in Figure 4. Then the user co-manipulated the robot using admittance control and aligned the drill tip with the center of the first virtual sphere. Once the alignment was assessed as adequate, the position of the drill tip was registered in {R} and the center of the sphere was registered in {H}. Then the user co-manipulated the robot to the next calibration sphere and registered the second pair of points. This step was repeated for all six calibration points, and finally, the homogeneous transform of the robot–HoloLens matrix (${}_{H}^{R}T$) was calculated by solving the orthogonal Procrustes problem [18]. The flowchart of the procedure is shown in Figure 4.

### 2.4. Experimental Procedure

**Figure 5:**
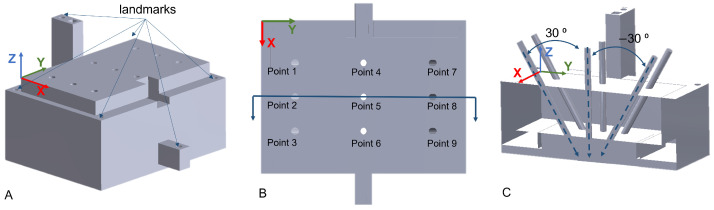
(**A**) Side view of the phantom and the six landmarks used for phantom registration; (**B**) top view of the phantom with the nine target ID used during the experimental validation; (**C**) section view of the phantom along the line in (**B**).

User experiments were carried out to evaluate the positioning accuracy of the AR-based robotic system involving three participants. A 3D-printed phantom was used for quantitative assessment and validation. The phantom was filled with Eco-flex with nine holes as reference drilling targets, as shown in Figure 5. The first three targets were oriented at 30${}^{\circ }$ about the X axis. Targets 4–6 were vertical, while the last three targets were at −30${}^{\circ }$ about the X axis. The users were standing on the left side of the phantom with targets 1–3 being closest to them. The phantom had a depth of 30 mm along the Z axis. The user experiments were composed of three consecutive steps, which are further discussed in the following. The calibration procedure and the positioning of the robot are also shown in the supplementary material Video S1.

1. Registration of the ground truth targets in {R}.
2. Spatial calibration of HoloLens and robot, as described in Section 2.3.
3. Positioning of the drill to the nine targets using the AR interface.

#### 2.4.1. Registration of the Ground Truth

Before the user experiments, the phantom {Ph} to robot {R} transformation (${}_{R}^{Ph}T$) was determined by point-to-point rigid registration. This registration was necessary to compare the desired and the actual pose of the nine targets in {R}. A similar calibration approach as described in Section 2.3 was used. The six landmarks, shown in Figure 5, were touched with the drill tip using admittance control, and the pose of the drill tip in {R} was recorded. Once all six defined target landmarks were registered, the transformation matrix ${}_{R}^{Ph}T$ was computed. Using the established transformation poses of the nine desired targets in {Ph} were then converted to {R}.

#### 2.4.2. Positioning of the Drill Using AR

**Figure 6:**
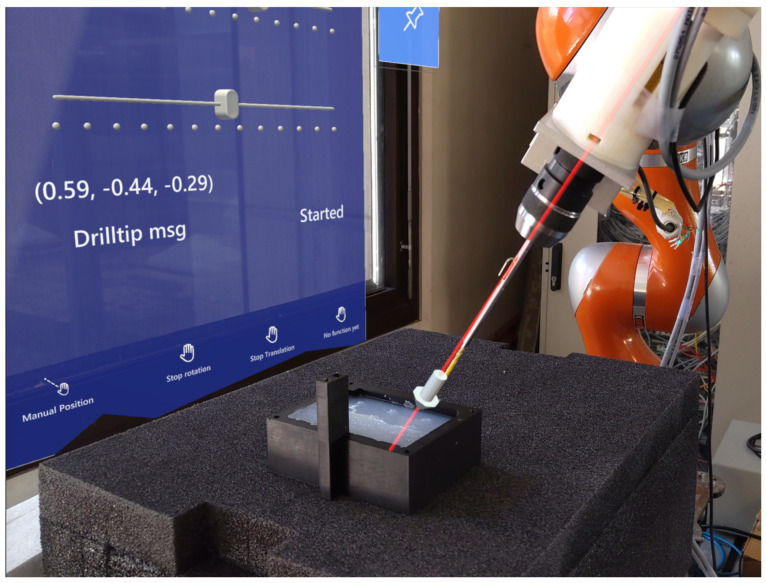
The positioning of the robot using the AR interface. After defining the pose with the AR arrow, the robot was moved using the GUI and aligned with the arrow.

During the experiments, the accuracy and performance of the proposed system were assessed. Following the calibration of the robot and HoloLens, the participants placed the virtual arrow to the targets sequentially (from Point 1 to Point 9) on the phantom and then controlled the robot to the defined target pose using the AR interface, as shown in Figure 6.

### 2.5. Evaluation Metrics

Following the user experiments, the assessment of the system was composed of four metrics, namely (1) the accuracy of the robot–HoloLens calibration, (2) the effect of the human factor, (3) the accuracy of the overall positioning of the drill, and (4) the execution time of defining an entry point. During the evaluation of the results, the ground truth values were affected by the calibration error between the phantom and {R}. All metrics were investigated in the robot coordinate frame {R}. The calculations of the aforementioned metrics are discussed in the following subsections.

#### 2.5.1. Evaluation of Calibration Accuracy

The accuracy of the calibration between the HoloLens and robot was assessed by transforming the six calibration points from {H} to {R} using ${}_{H}^{R}T$. These coordinates were then compared to the locations of the calibration points as measured by the robot in {R} and the error was calculated along the *x*, *y*, and *z* axes by

(2) $$E=|P_{R_{i}}{-}_{H}^{R}T\cdot P_{H_{i}}|,i=1\ldots 6$$

where $P_{R_{i}}$ is the ith calibration point in {R} and $P_{H_{i}}$ is the ith calibration point in {H}. Furthermore, the normalized mean error was calculated by

(3) $$E_{norm}=\frac{\sum_{n=1}^{6}\sqrt{E_{x_{n}}^{2}+E_{y_{n}}^{2}+E_{z_{n}}^{2}}}{6}$$

where $E_{x}$, $E_{y}$, and $E_{z}$ are the errors along the *x*, *y*, and *z* axis in {R}, respectively.

#### 2.5.2. Evaluation of Human Factor

Independently from the calibration error, the human factor error also affected the participant’s performances. This human factor error is a combination of how well the user can perceive and position the AR cues, and the influence of hardware limitations on his or her performance, such as the 3D rendering error and resolution of the HoloLens. In some cases, it was difficult to make precise adjustments to the AR-rendered objects using hand gestures, which resulted in a slight offset from the intended pose.

The human factor error was evaluated based on how well the users could estimate the relative distance between the target points when setting drilling poses. Point 1 was used as the reference point and the distances to the other target points were calculated based on the set targets by the users. These distances were then compared to the ground truth distances on the phantom.

#### 2.5.3. Evaluation of Positioning Accuracy

When evaluating the positioning accuracy of the system, the *z* coordinates were not taken into account, as it was noticed that the users tended to place the tip of the AR arrow slightly higher than the entry point on the surface of the phantom. This behavior is also expected during surgery when the user may feel safer doing so. The resulting drill tip positions were projected along the centerline of the drill onto the surface of the phantom. The overall performance of the target pose error was evaluated based on the positioning of the AR arrows at the nine targets of the phantom. The overall target pose error was calculated as the distance between the ground truth entry point and the projected entry point on this 2D plane using

(4) $$E_{D}=\sqrt{{\left(x_{ref}-x_{P}\right)}^{2}+{\left(y_{ref}-y_{P}\right)}^{2}}$$

where $x_{ref}$ and $y_{ref}$ are the references, while $x_{P}$ and $y_{P}$ are the projected *x* and *y* coordinates. To evaluate the orientation, the error was calculated between the measured and the reference Euler angles. The estimation of the correct angle was assessed by comparing the resulting orientation to the ground truth and calculating the error in the Euler angle about the *x* axis.

Furthermore, after defining the pose of the entry point and moving the robot using the AR interface, the user drilled through the phantom. The pose error was also evaluated based on the distance error on the bottom surface of the phantom, called the drill target error as

(5) $$E_{Dt}=d\cdot tan\left(E_{R_{x}}\right)+E_{D}$$

where $E_{Dt}$ is the drill target error, *d* is the depth of the phantom, $E_{R_{x}}$ is the rotation error about the *x* axis, about which the targets were rotated, and $E_{D}$ is the distance error on the top surface of the phantom.

Besides the positioning accuracy, the execution time was also assessed, by measuring the time it took to define the entry point. Before each target, the AR arrow was moved ±500 mm above the phantom. When positioning the arrow, the time was measured between grabbing the arrow and the time when the arrow was considered as correctly placed at the entry point.

## 3. Results

Three users with little experience with AR participated in the experiments. Table 1 summarizes the resulting robot–HoloLens calibration error, which was found to vary between 0.08 and 6.39 mm, where the largest error was measured for User 2 along the *z* axis. In all other cases, the maximum errors were within 4 mm. The normalized mean error $E_{norm}$ was 3.43±1.35 mm, 5.18±1.2 mm, and 2.23±0.95 mm for User 1, User 2, and User 3, respectively.

**Figure 7:**
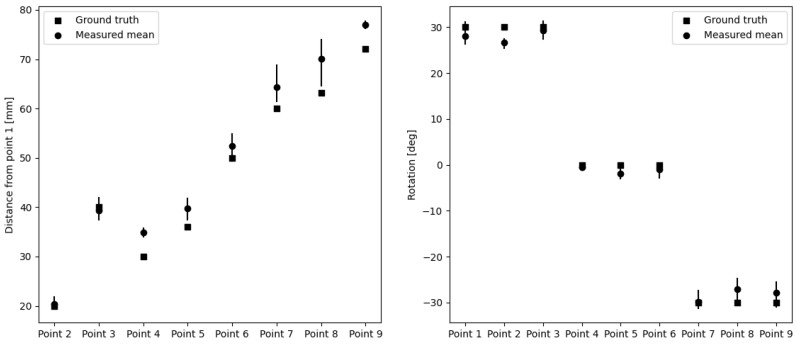
The results of the human factor error assessment. **Left.** The distances are measured with respect to Point 1. **Right.** The rotations are measured about the *x* axis. The vertical lines together with the mean values show the minimum and maximum values of the three users.

To evaluate the human factor error and the consistency of the users, the distances between the indicated entry points were calculated with respect to Point 1. Table 2 summarizes the resulting errors. The maximum distance error was 10.78 mm for Point 8, while the mean distance error was 3.36±2.36 mm. The holes in the phantom were only rotated about the horizontal *x* axis. Therefore, the rotation errors were only calculated about the *x* axis and are summarized in Table 3. The maximum rotation error was $-5.29^{\circ }$ for Point 8, while the mean rotation error was $0.47\pm 1.86^{\circ }$. Figure 7 compares the ground truth with the measured distances and rotations.

**Figure 8:**
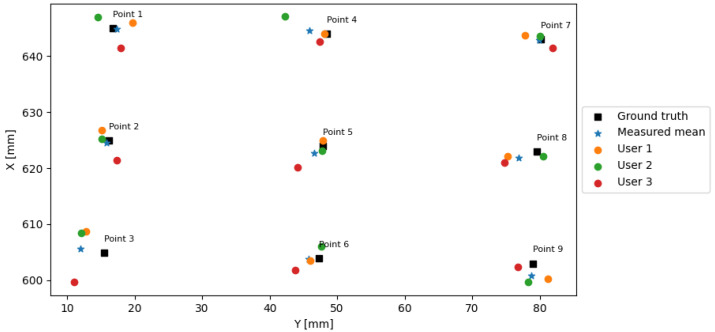
The results of the comparison of the ground truth values, the individual, and the mean positions of the drill tip by using the AR arrow. The positions were measured in {R}.

The final target pose errors consisting of the position and the angular accuracy during the user experiments are summarized in Table 4. The users could position the drill tip using the AR interface within a distance $E_{D}$ of 6.93 mm with a mean distance of 3.05 mm from the ground truth. The rotation error was below 8${}^{\circ }$ with a mean of 2.02 ± 0.6${}^{\circ }$. Figure 8 depicts the 2D positions of the ground truth points and the positions defined by each user together with the mean positions. Following the drilling using the defined entry point pose, the drill target error ranged from 0.13 to 8.62 mm with a mean of 3.83 for 30 mm depth, while it ranged from 0.24 and 11.13 mm with a mean of 4.77 mm for a depth of 60 mm.

Table 5 reports the execution times of the nine points for the users. The mean execution time for the entry points with 30${}^{\circ }$ angle was 30.17 s, while the mean time for the entry points with −30${}^{\circ }$ angles was 6 s. On average, the users could position the vertical entry points faster, with a mean time of 20.51 s. It is believed that it was easier for the user to assess whether the arrow was vertical compared to when it was at a certain angle.

## 4. Discussion

In this paper, an AR-based robot control scheme was developed for pedicle screw placement. The objective of the proposed approach was to simplify the complex robot control in spine surgery for clinical users and to allow a fast way for intra-operative adjustment of the surgical plan based on the updated patient anatomy or position. The system consists of a robotic arm, a HoloLens for AR visualization and interaction, and a PC for communication and control. The system was evaluated via a user study, where three participants executed a positioning task by placing a virtual arrow on a silicon phantom to define the drill tip pose at nine pre-defined reference entry points. The mean distance and the rotation error from the ground truth points for the three users were 3.05 mm and 2.15${}^{\circ }$, respectively, as shown in Table 4. The overall maximum error of the three users was 6.93 mm for translation and 8${}^{\circ }$ for rotation. These errors are smaller than the mean error reported by Ferraguti et al. [14], who described a mean translation error of 15.18 mm when positioning a needle in AR-assisted percutaneous nephrolithotomy. Overall, when positioning the drill tip using the AR interface, the error was propagated of the calibration, the human factor, and the ground truth registration error.

As the normalized mean calibration errors showed, there was almost a 3 mm difference between User 3 and User 2, who performed the best and the worst, respectively. The users attempted to precisely align the tip of the drill with the center of the virtual spheres, though having limited experience with the HoloLens. When the user moved his/her head, the AR spheres in the 3D hologram slightly moved as they were re-rendered for the new viewpoint. This movement of the holograms made it challenging to perfectly align the real and virtual points in 3D space. Guo et al. [19] proposed an online calibration method to find the transformation matrix between the world coordinate frame and the HoloLens frame using a Micron Tracker and visual markers on a calibration box. The mean calibration error of their proposed method was 6.83 mm. Ferraguti et al. [14] also used visual markers to calibrate the world coordinate frame with the HoloLens frame, with a calibration error of 15.8 mm. Although the proposed calibration method in this work might be more user-dependent, the mean error is significantly lower than in the aforementioned two studies, reaching only 3.61 mm. However, it could still be improved as Tu et al. [15] could achieve an RMS calibration error of 1.64±0.48 mm, although, when using an optical camera. The proposed calibration approach in this work omits the need for such external tracking devices, which can be influenced by the line-of-sight and environmental light quality. It also reduces the hardware requirements of the overall system. However, despite the promising results, the calibration method is influenced by the instability of the depth sensing of the HoloLens, which introduces a deviation of 3.38 mm at a 70 cm distance according to [20].

When positioning the AR arrow to define the entry point for the drill, besides the calibration error, the human factor also affected the overall performance. This human factor error depends on the user’s assessment of the correct pose in 3D. Furthermore, the HoloLens was still tracking hand gestures when the user wanted to release the arrow. This resulted in slight movements away from the intended point. The human factor error was evaluated based on the consistency of positioning, by analyzing the translation and rotation error between the entry points. This factor was independent of the calibration error as the relative distances and rotations were measured. As Table 2 shows, the overall mean distance error was 3.36 mm. The smallest mean errors of the three users were 0.42 and −0.72 mm in Point 2 and Point 3, respectively. These points only had a translation along the *x* axis with respect to Point 1. The larger the distance was from the reference, the larger the error became, except in Point 4, where the mean error was 4.92 mm, while being the second closest point. The entry points farther from the reference were also farther from the user. The depth perception of users at larger distances may vary when looking at the AR content, which could further affect the human factor error when positioning at larger distances. The rotation errors were relatively small with a mean of 0.47${}^{\circ }$ as summarized in Table 3. The rotation error was the smallest when the entry points were vertical, with an absolute mean error of 1.12${}^{\circ }$. The larger errors were measured when the entry points were tilted 30${}^{\circ }$, for points closer to the users. The mean error dropped to 1.99${}^{\circ }$. The rotation error was only assessed about the *x* axis for the human factor error as the entry points on the phantom were only rotated about the local *x* axis. However, the phantom was not perfectly aligned with the robot frame, thus a more thorough evaluation of the drilling target pose error would be worthwhile.

After defining the pose at the entry point and positioning the robot using the AR interface, the drilling through the phantom was performed and the error on the bottom surface of the phantom, called drill target error was assessed. This drill target error was the combination of the entry point error and the rotation error. Due to the rotation component, the error was expected to increase with increasing depth. Therefore, besides evaluating the error for the 30 mm depth of the phantom, Table 4 shows the error also for 60 mm, double depth. As expected, the mean error was 1 mm larger in the case of a 60 mm depth, while the maximum error was 4.22 mm larger.

The AR interaction was based on hand gestures and voice commands, where the user could set drilling parameters and move the robot using a dedicated GUI, and place a virtual arrow to define the drill tip pose. To improve the positioning, the user could lock 5 DOF out of the total 6, thus, only manipulating the remaining free DOF, e.g., translate along a single axis or rotate about a single axis. This was beneficial, e.g., when the user assessed the correct position along the *y* axis and only wanted to adjust the tip position along the *x* axis.

In this paper, only a user study was conducted to assess its technical feasibility, its system accuracy, and related errors. This work has several limitations. First, only three users participated with no medical background due to limited time and access to clinical partners. Second, the current laboratory setting is a rather optimal condition when compared to that of in the OR. Based on the experience, with uniform background, the accuracy of depth drops. Since the current work proposes the technical feasibility of such AR-based HRI, the evaluation was carried out in an ideal (less uniform and dynamic) environment than an operating room. Therefore, before deploying to the OR, the experimental assessments of the system with a cohort of subjects, including clinicians, are foreseen in the future study and a simulated OR environment. The accuracy of the positioning might be negatively affected by non-ideal circumstances. Furthermore, while the spatial calibration without an external tracking device seems promising, it is also user-specific as it depends on how precisely the user aligns the drill tip with the calibration spheres. Further methods could be considered in the future that might also be easier to perform in the OR, for example, a quick bootstrap procedure to complete the calibration step. Lastly, the overall ergonomics of the system were not assessed, as it was out of the scope of this research. However, it is important to note that wearing the HoloLens for a longer time and working at relatively close distances might introduce fatigue and eye strain for the user. This will require a usability study focusing on subjective evaluation. In addition, besides defining the pose of the entry point and analyzing its accuracy, further evaluation would be directed to analyze drilling trajectory accuracy on the bony tissue from a cadaver or animal models.

## 5. Conclusions

In summary, the developed system offers an intuitive interaction between the surgeon and surgical robot when performing surgical actions, e.g., pedicle screw placement and intra-operative surgical plan adjustment. The surgical plan can be made and adjusted by a surgeon on-site. Meanwhile, thanks to the real-time interaction, any changes made by the surgeon in AR can be immediately mapped to the robot side for execution. Such a promising augmented reality-based robot control scheme has great potential to change the paradigm of means of the controlling surgical robot for the end users. A major advantage of the proposed system is that no external tracking device was used to calibrate the system to find the transformation matrix between the HoloLens and the robot frame. In cases of external optical trackers, occlusions of the visual markers interrupt the operation. This can occur in a crowded OR. The proposed system eliminates this risk of occlusions. Furthermore, this simplifies the overall system that needs to be introduced in an operating room. The experiments showed that the readjustment of the position of the arrows took 26.56 s on average in a free-touch fashion. This allows a fast intra-operative update of the surgical plan and positioning of the robot to the desired pose. The overall position error might not be adequate for direct AR-based robotic-assisted PSP due to the positioning errors measured. However, the entry point could be fine-tuned by a vision-based system that identifies the anatomic landmarks. Such fast positioning of the pedicle screw to the region of interest could further enhance the performance of robotic-assisted PSP and reduce the operation time. Consequently, the proposed system would establish a new approach to the AR–robot interaction for medical applications in the OR.

## Figures and Tables

**Table 1:** Results of the assessment of the calibration accuracy. The values for each user are calculated from the six calibration points. All errors are in mm in {R}.

	User 1			User 2			User 3		
	$E_{x_{1}}$	$E_{y_{1}}$	$E_{z_{1}}$	$E_{x_{2}}$	$E_{y_{2}}$	$E_{z_{2}}$	$E_{x_{3}}$	$E_{y_{3}}$	$E_{z_{3}}$
Min	0.71	0.81	0.79	0.08	0.23	2.04	0.23	0.21	0.68
Max	1.28	3.69	3.94	3.18	3.67	6.39	1.82	2.75	1.69
Mean	0.99	2.15	2.35	1.69	1.96	3.99	0.97	1.49	1.12

**Table 2:** Results of the assessment of the human factor error in translation. $D_{ref}$ are the reference distances, while $E_{D_{m}}$ is the error in the measured distance of the user’s arrow placement. The distances are with respect to Point 1 and all values are in [mm].

Point	$D_{\mathrm{ref}}$	$E_{D_{m}}$ min	$E_{D_{m}}$ max	$E_{D_{m}}$ Mean
Point 2	20	−0.49	1.77	0.42
Point 3	40	−2.54	1.98	−0.72
Point 4	30	3.99	5.7	4.92
Point 5	36.06	1.44	5.76	3.73
Point 6	50	−0.59	4.83	2.38
Point 7	60	1.43	8.75	4.37
Point 8	63.25	1.41	10.78	6.90
Point 9	72.11	4.17	5.62	4.84

**Table 3:** Results of the assessment of the human factor error in rotation. $R_{ref}$ is the reference rotation about the horizontal *x* axis, while $E_{R_{m}}$ are the measured rotation errors about the *x* axis. All values are in degrees.

Point	$R_{\mathrm{ref}}$	$E_{R_{m}}$ min	$E_{R_{m}}$ max	$E_{R_{m}}$ Mean
Point 1	30	−1.08	3.71	1.95
Point 2	30	2.51	4.56	3.31
Point 3	30	−1.33	2.59	0.71
Point 4	0	0.35	0.83	0.53
Point 5	0	1.04	3.04	1.94
Point 6	0	−0.06	2.83	0.91
Point 7	−30	−2.61	1.26	−0.11
Point 8	−30	−5.29	−0.69	−2.89
Point 9	−30	−4.44	0.98	−2.11

**Table 4:** Assessment of positioning accuracy. $E_{D}$ represents the distance error between the ground truth and the measured drill tip position on the phantom surface. $E_{R_{x}}$, $E_{R_{y}}$, and $E_{R_{z}}$ are the Euler angle errors along the *x*, *y*, and *z* axis, respectively. $E_{Db}^{30}$ and $E_{Db}^{60}$ represent the drill target errors for depths of 30 and 60 mm, respectively. All values are measured in {R}.

	User 1			User 2			User 3		
	$\min_{1}$	$\max_{1}$	${\mathrm{mean}}_{1}$	$\min_{2}$	$\max_{2}$	${\mathrm{mean}}_{2}$	$\min_{3}$	$\max_{3}$	${\mathrm{mean}}_{3}$
$E_{D}$ [mm]	0.37	4.72	2.54	0.62	6.91	2.68	1.75	6.93	3.93
$E_{R_{x}}$ [${}^{\circ }$]	0.15	8.00	2.84	0.59	7.39	3.10	0.28	6.43	2.30
$E_{R_{y}}$ [${}^{\circ }$]	0.29	4.92	2.25	0.40	2.75	1.21	0.02	2.97	1.32
$E_{R_{z}}$ [${}^{\circ }$]	0.16	6.21	2.72	0.14	4.48	1.94	0.15	4.21	1.45
$E_{Db}^{30}$ [mm]	0.13	8.62	3.14	0.52	7.55	3.57	2.37	7.43	4.76
$E_{Db}^{60}$ [mm]	0.24	12.84	4.00	0.62	11.13	4.71	2.01	9.68	5.60

**Table 5:** Results of the assessment of the execution time.

	User 1 [s]	User 2 [s]	User 3 [s]	Overall Mean [s]
Point 1	61.47	28.13	18.02	35.87
Point 2	21.09	21.47	28.12	23.56
Point 3	38.05	33.46	21.69	31.07
Point 4	24.46	10.2	14.02	16.23
Point 5	28.37	10.63	26.18	21.73
Point 6	32.37	9.63	28.75	23.58
Point 7	31.93	15.06	20.53	22.51
Point 8	36.45	11.09	34.28	27.27
Point 9	37.59	17.02	30.08	28.23
Mean	34.64	17.41	24.63	26.56

## Data Availability

Not applicable.

## Supplementary Materials

The following supplementary material is available online at www.mdpi.com/xxx/s1: Video S1: AR-based robot control: calibration procedure and robot positioning.

## Abbreviations

The following abbreviations are used in this manuscript:

AR	augmented reality
DOF	degrees of freedom
GUI	graphical user interface
HMD	head-mounted displays
HRI	human–robot interaction
MR	mixed reality
OR	operating room
PSP	pedicle screw placement
UDP	user datagram protocol
